# Effect of Dual Endothelin Receptor Antagonist on a Retinal Degeneration Animal Model by Regulating Choroidal Microvascular Morphology

**DOI:** 10.1155/2021/5688300

**Published:** 2021-11-19

**Authors:** Xiaowei Zhu, Xuming Lin, Ying Xu, Naiyang Li, Qing Zhou, Xiaowei Sun, Yuanbin Li

**Affiliations:** ^1^Department of Ophthalmology, Zhongshan City People's Hospital, Zhongshan, Guangdong, China; ^2^Department of Ophthalmology, First Affiliated Hospital of Medical College of Jinan University, Guangzhou, Guangdong, China; ^3^Department of Ophthalmology, The Affiliated Yantai Yuhuangding Hospital of Qingdao University, Yantai, Shandong, China; ^4^Guangdong-Hongkong-Macau Institute of CNS Regeneration, Jinan University, Guangzhou, China

## Abstract

**Objective:**

Clinical studies have found that increasing levels of plasma endothelin-1 (ET-1) might inhibit choroidal blood flow (BF) and promote choroidal vasoconstriction. This study was designed to investigate ET-1 levels and its effect on choroidal microvascular morphology in a retinitis pigmentosa (RP) animal model.

**Methods:**

Mice with retinal degeneration (rd10) were intragastrically administered bosentan, a dual endothelin receptor antagonist. We detected plasma ET-1 levels using an enzyme-linked immunosorbent assay (ELISA) kit at P14, P21, and P28 and evaluated ET-1 expression in RPE/choroid/sclera complexes using western blot and whole mount immunofluorescence staining at P28. Retinal thickness was measured using hematoxylin and eosin (H&E) staining at P28. At the same time, we also estimated choroidal microvascular densities using vascular luminal casting with a scanning electron microscope (SEM).

**Results:**

Plasma ET-1 levels were increased significantly in rd10 mice at P21 (65.48 ± 24.83 pg/ml) and P28 (85.89 ± 20.23 pg/ml) compared with C57BL/6J mice at P21 (33.52 ± 16.33 pg/ml) and P28 (42.38 ± 17.53 pg/ml); the expression of ET-1 was also upregulated in RPE/choroid/sclera complexes at P28. Bosentan inhibited ET-1 expression in plasma (*P* < 0.05) and RPE/choroid/sclera complexes at P28 in rd10 mice. Choroidal microvascular densities were decreased in rd10 mice, and bosentan could weaken these changes.

**Conclusion:**

Plasma and local ET-1 was elevated in an animal model of RP, suggesting that it likely participates in the pathological progression of retinal degeneration and may thus provide a new intervention target. ET-1 blockade might exert its protective effect by elevating choroidal microvascular density via inhibition of ET-1.

## 1. Introduction

Retinitis pigmentosa (RP), a common cause of blindness in working age people [1], is a well-known hereditary disease with >200 gene variations; it is characterized by nyctalopia (night blindness) and concentric visual-field defects [2, 3]. Gene therapy offers a promising way to correct this genetic mutation; however, there are many limitations and challenges including that only a few RP mutations can be accurately treated and treatment usually has limited efficacy [4]. Importantly, pharmacological approaches that target the potential pathophysiological mechanisms of RP might supply alternative means of treatment.

A growing number of clinical studies have demonstrated that retinal and choroidal blood flow (BF) decreases with the progression of RP [5, 6], suggesting that such decreases might lead to ischemia of the retina, especially in photoreceptors. Hemodynamic research has shown increased levels of the potent vasoconstrictor endothelin-1 (ET-1) in RP patients [7], which is negatively correlated with retinal and choroidal BF in early and advanced stages of RP [8].

ET-1, which is mainly secreted by vascular endothelial cells (ECs), can constrict vessels, promote the proliferation of vascular smooth-muscle cells, and remodel vasculature. It mediates these physiological functions mainly through two subtype receptors of ET_A_ and ET_B_, which are also expressed in human and animal choroids [9, 10]. Researchers have reported lower intraocular pressure in glaucoma animal models after administration of the ET_A_ selective antagonist 97-139 or ET_B_ selective receptor antagonist sarafotoxian S6c and the retinal and choroidal BF were increased in glaucoma patients by taking nonselective receptor antagonist bosentan [11, 12]. Bosentan was a nonpeptide orally active dual ET_A_ and ET_B_ antagonist chemically named 4-tert-butyl-N-[6-(2-hydroxyethoxy)-5-(2-methoxyphenoxy)-2-(2-pyrimidinyl)-4-pyrimidinyl] benzenesulfonamide mainly used as a first-line treatment option for pulmonary artery hypertension [13]. For this reason, we assumed that ET-1 may participate in the pathological progress of RP and ET-1 blockade using bosentan might slow it down by regulating the choroidal BF.

Animal research using high-resolution magnetic-resonance imaging (MRI) to test retinal and choroidal BF has revealed that retinal BF decreases significantly and choroid BF remains unchanged in RP [14], which contradicts the abovementioned clinical findings. High-resolution MRI might not distinguish subtle differences in choroidal BF and microvasculature for the tiny microvascular tissue in the choriod of mice or other small animals. Vascular luminal casting conserves microvascular morphology by using a scanning electron microscope (SEM) [15], which can detect early deterioration of choroidal microvasculature in animals with RP. Therefore, we applied the vascular luminal casting technique in order to examine choroidal microvascular morphology in a RP mouse model.

Because there are no reports on ET-1 and its effect in RP animal models, this study was designed to investigate the pattern of plasma ET-1 in rd10 mice, a typical RP model with a nonsense mutation of the pde6*β* gene, using bosentan, a dual endothelin receptor antagonist, to determine whether ET-1 blockade could slow down the progression of RP by regulating choroidal microvascular morphology in this model.

## 2. Materials and Methods

### 2.1. Animals and FA Treatment

rd10 mice were provided by Professor Ying Xu; these mice were purchased from Jackson Laboratory (Bar Harbor, ME, USA), and C57BL/6J mice were purchased from Beijing Vital River Laboratory Animal Technology Co., Ltd. (Beijing, China). All mice were maintained in specific pathogen-free (SPF) laboratories at Yantai Yuhuangding Hospital (YYH; Yantai, China), and 34 C57 mice and 46 rd10 mice were used in this experiment. This study was approved by the Animal Ethics Committee of YYH and was performed in accordance with the Association for Research in Vision and Ophthalmology (ARVO) Statement for the Use of Animals in Ophthalmic and Vision Research. There were three groups in this study: a C57BL/6J control group, a rd10 group that received phosphate-buffered saline (PBS) intragastrically (i.g.), and a rd10+bosentan group that received 100 mg/kg bosentan (#1076115; Sigma-Aldrich, St. Louis, MO, USA) i.g. daily from P7 to P28. We randomly divided rd10 mice into the rd10 or rd10+ bosentan groups. Mice were anesthetized with isoflurane before intragastric administration of bosentan. And, mice were anesthetized with avertin (#T48402; Sigma-Aldrich, USA) before vascular luminal casting at P28. Mice were sacrificed by intraperitoneal (i.p.) injection of 200 mg/kg sodium pentobarbital (#P-010; Sigma-Aldrich, USA) for detection of plasma ET-1 levels at P14, P21, and P28, and the eyeballs were enucleated for H&E staining, immunofluorescence, and western blot at P28.

### 2.2. Plasma ET-1 Determination

After sacrificing the mice, we disinfected their periocular fur with 75% alcohol. The eyes were enucleated, and 0.5 ml blood from the intraorbital vessels was extracted into 1.5 ml anticoagulant tubes (Eppendorf, Hamburg, Germany). We shook the tubes several times to mix the blood, centrifuged the samples at 4°C for 20 min, extracted the supernatant (plasma), and stored the samples at −80°C. Levels of plasma ET-1 were analyzed using an enzyme-linked immunosorbent assay (ELISA) kit (Cusabio Biotech, Wuhan, China) as per the manufacturer's instructions.

### 2.3. Western Blotting

After enucleating the eyeballs and cutting off conjunctivae, the RPE/choroidal/scleral complexes were isolated by removing the corneas, lenses, and retinas. The proteins in these complexes were homogenized in RIPA lysis buffer (Beyotime Institute of Biotechnology, China). The protein concentration was determined, and 20 *μ*g of proteins from every group was used for the following procedures, as previously reported [16]. The primary antibody of mouse anti-endothelin 1 antibody (1∶1000; ab2728, Abcam, MA, USA) and the secondary antibodies of anti-mouse IgG antibody (1∶1000; 7076#, CST, USA) were used, and the gray intensity was estimated using a ChemiDoc™ MP Imaging System (BioRad, USA).

### 2.4. Immunofluorescence Staining on RPE/Choroidal/Scleral Whole Mounts

Eyeballs were fixed in 1% PFA (paraformaldehyde) for 1 h to fix choroidal vasculature. We washed away the PFA with PBS and produced the RPE/choroidal/scleral whole mounts by separating the conjunctivae, corneas, lenses, and retinas. The mounts were incubated for 2 h at 37°C, and the RPE was washed away with PBS. The mounts were fixed in 1% PFA for 1.5 h again. After washing the PFA, the mounts were blocked using 0.5% Triton X-100/5% BSA overnight and incubated with the primary antibodies of ET-1 (1∶100; ab2728, Abcam, MA, USA) and CD31 (1∶100; MAB1398Z, Millipore, MA, USA) for 2 days at 4°C. The secondary antibodies of anti-mouse IgG antibody and anti-hamster IgG antibody (1∶800; Alexa Fluor® 488 Conjugate and Alexa Fluor® 647 Conjugate, CST, USA) were added for 1 h. The mounts were photographed using a Zeiss confocal microscope (LSM710, Carl Zeiss).

### 2.5. Cardiac Perfusion and Vascular Luminal Casting

Mice received i.p. injections of 1.2% avertin (0.15 ml/10 g) at P28. After anesthesia, we enucleated the left eyeballs for H&E staining and fixed the mice on an anatomical table. Then, cardiac perfusion was performed as previously described [17].

Batson's #17 Anatomical Corrosion Kit resin (Polysciences, Inc., Warrington, PA, USA) was injected into the left-ventricular catheter until viscous light-yellow fluid flowed out of the right-ventricular catheter. Mice were kept at room temperature for 4 h until the casting agent solidified. We extracted the right eyeballs and made two corneal-limbus incisions on the nasal and temporal sides to mark the position. We then immersed the eyeballs in 20× volume of 40% NaOH solution for 3 days. After repeated washes with PBS, we cut off the anterior segments of the eyes under a stereomicroscope. The retinal vessels were carefully separated, and the choroidal microvascular layer was exposed. The castings were dehydrated by 70%, 85%, 95%, and 100% ethanol. After mounting, we vacuum-sprayed choroidal castings with gold coating. The choroidal microvascular layer was observed with a scanning electron microscope (SEM; Hitachi, S-520, Japan; magnification, 500×). We examined the nasal and temporal parts of each castings, and the detected locations were two diameter of optic papillae (PD) away from the optic papillae.

### 2.6. Hematoxylin and Eosin Staining

We fixed the left eyeballs in formalin overnight, embedded them in paraffin, and cut them into 3 *μ*m sections that were collected on glass slides. Next, we washed the sections in deionized water and stained them in hematoxylin buffer for 15 min. After another wash, sections were incubated with 1% eosin solution for 15 s. Finally, we rehydrated the slices in alcohol gradient and mounted them with coverslips. Images were acquired with a Leica DM4000 microscope (Leica, Wetzlar, Germany); retinal thickness was analyzed 200 *μ*m far away from the optic-nerve head [18]. 4 eyes of each group and 3 slides of each eye were used for analysis.

### 2.7. Statistical Analysis

All data are presented as mean ± standard deviation (SD). We used a two-tailed Student's *t*-test and one-way analysis of variance (ANOVA) to analyze differences between the control and treatment groups. SPSS version 19.0 (IBM Corp., Armonk, NY, USA) was used for all analyses.

## 3. Results

### 3.1. ET-1 Was Elevated in rd10 Mice

**Figure 1:**
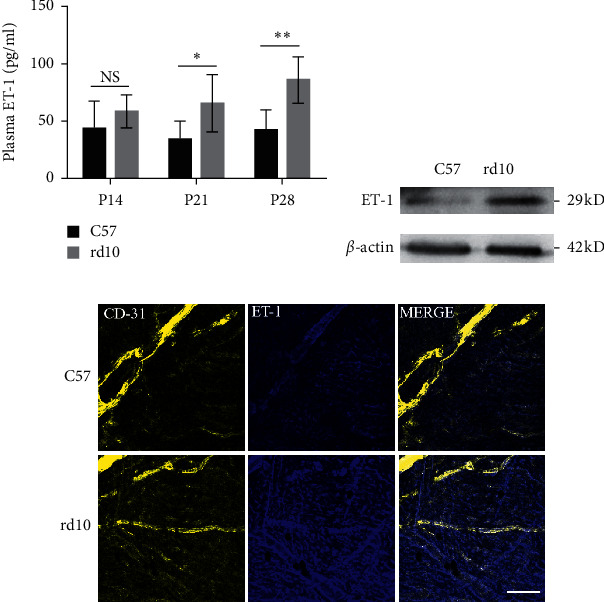
(a) Plasma ET-1 levels (pg/ml) in C57 and rd10 mice at P14, P21, and P28 (*n* = 6). Western blot (b) and immunofluorescence staining (c) showed that ET-1 expression was elevated in RPE/choroidal/scleral complexes at P28 (*n* = 4). Scale bar: 50 *μ*m. ^∗^*P* < 0.05,^∗∗^*P* < 0.01, and ^∗∗∗^*P* < 0.001.

Plasma levels of ET-1 were increased in rd10 mice, while those in C57 mice remained relatively stable from P14 to P28 (Figure 1(a)). Levels were 95% higher at P21 (*P* < 0.05) and 103% higher at P28 (*P* < 0.01). Details are summarized in Table 1. We then determined whether ET-1 was upregulated in the local tissue of rd10 mice. Western blot data showed that ET-1 levels were visibly higher in RPE/choroidal/scleral complexes in rd10 mice than in C57 mice (Figure 1(b)). Immunofluorescence staining of RPE/choroidal/scleral whole mount showed that ET-1 levels were higher in rd10 mice and part of the cells were CD31/ET-1-positive which implied that except for the vascular endothelial cells, other cells might produce ET-1 or express its receptor in choroid.

### 3.2. Choroidal Microvasculature Was Degenerated in rd10 Mice as Revealed by Vascular Luminal Casting

**Figure 2:**
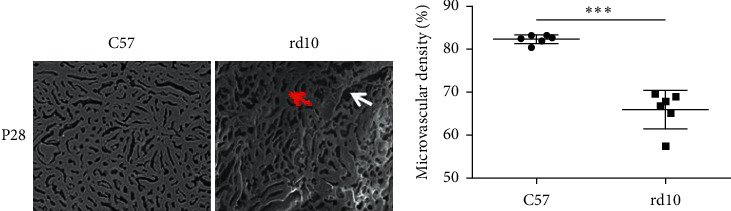
Choroidal microvascular density was significantly lower in rd10 mice than in C57 mice. Red arrow-indicated area was choroidal microvascular lumen, and white arrow-indicated area was the wall outside choroidal microvascular, or medium and larger, vessels (*n* = 6, magnification 500×). ^∗^*P* < 0.05,^∗∗^*P* < 0.01, and ^∗∗∗^*P* < 0.001.

In Figure 2, the black points or strips represent walls outside choroidal microvascular, or medium and larger, vessels, and the gray areas represent choroidal microvasculature lumens viewed with the SEM. The percentages of microvasculature lumen areas to the casting are calculated as microvascular densities. Choroidal microvascular densities were significantly lower in the rd10 group than in the C57 group (*P* < 0.001) at P28. More medium and large vessels appeared in the luminal casting of the rd10 group than that of the rd10+bosentan group.

### 3.3. Bosentan Inhibited ET-1 Expression and Alleviated Retinal Degeneration in rd10 Mice

**Figure 3:**
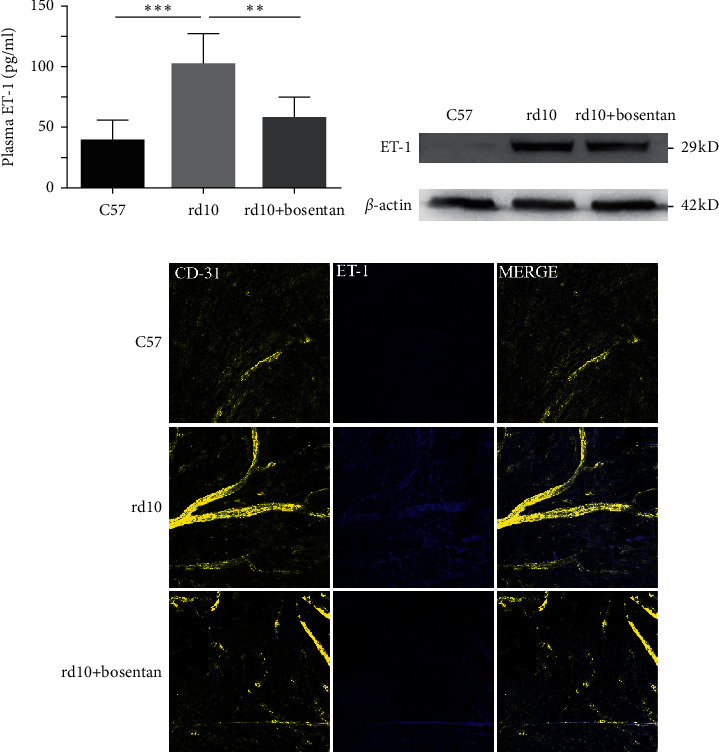
(a) Plasma ET-1 levels (pg/ml) in C57, rd10, and rd10+bosentan groups at P28 (*n* = 6). (b, c) ET-1 expression was also elevated in RPE/choroidal/scleral complexes at P28 (*n* = 4). Scale bar: 50 *μ*m. ^∗^*P* < 0.05,^∗∗^*P* < 0.01, and ^∗∗∗^*P* < 0.001.

We then used bosentan, a dual endothelin receptor antagonist, to determine whether it could reduce ET-1 levels and postpone the retinal degeneration. The elevated plasma ET-1 tendency of rd10 mice was inhibited by bosentan, showing significant disparity at P28 (*P* < 0.01; Figure 1(a)). Similarly, the expression of ET-1 was restrained by bosentan in RPE/choroidal/scleral complexes of rd10 mice (Figures 3(b) and 3(c)).

**Figure 4:**
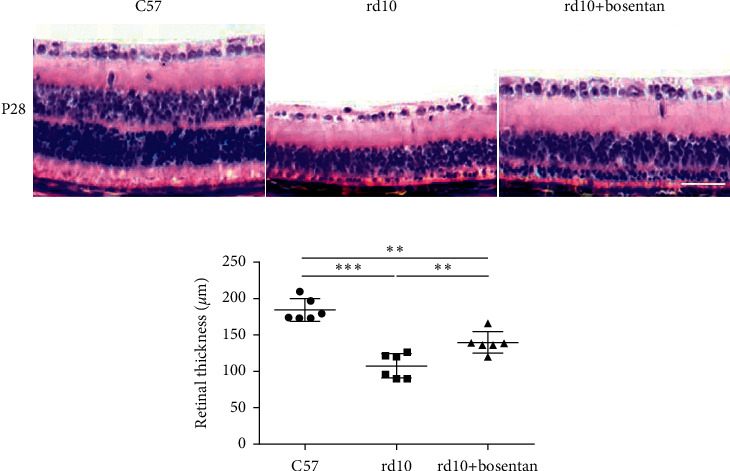
H&E staining results showed the retinal thicknesses of C57 mice, rd10 mice, and rd10 mice treated with bosentan at P28. The retinal thicknesses were increased significantly in the rd10+bosentan group compared to the rd10 group (*n* = 4). The scale bar was 50 *μ*m. ^∗^*P* < 0.05,^∗∗^*P* < 0.01, and ^∗∗∗^*P* < 0.001.

Retinas were thinner in the rd10 group than in the C57 group at P28. However, the rd10+bosentan group showed thicker retinas than the rd10 group (*P* < 0.01; Figure 4). These data indicated that bosentan might be able to delay retinal degeneration.

### 3.4. Bosentan Prevented Choroidal Microvascular Degeneration in rd10 Mice

**Figure 5:**
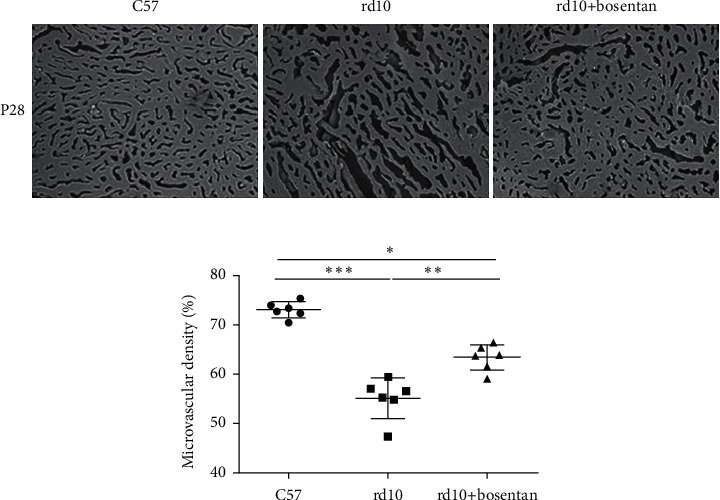
Choroidal microvascular densities were significantly decreased in rd10 mice compared to C57 mice and elevated by bosentan treatment at P28 by scanning electron microscopy (*n* = 6, magnification 500×). ^∗^*P* < 0.05,^∗∗^*P* < 0.01, and ^∗∗∗^*P* < 0.001.

Choroidal microvascular densities were significantly lower in the rd10 group than in the C57 (*P* < 0.001) and rd10+bosentan groups (*P* < 0.01), slowing down the progression of choroidal microvascular atrophy (*P* < 0.01; Figure 5). Bosentan might alleviate retinal degeneration by regulating the choroidal microvascular morphology.

## 4. Discussion

Our data demonstrated that ET-1 levels of the plasma and RPE/choroidal/scleral complexes were increased in rd10 mice, which was consistent with the results of clinical studies [7, 8]. Furthermore, we found that bosentan could promote choroidal microvasculature dilation, possibly by decreasing plasma and local ET-1. This underscores the possibility of regulating the choroidal microvasculature as a new strategy for alleviating retinal degeneration.

In the progression of retinal degeneration, the thinning of the retinal vessels causes microvascular abnormalities and BF impairment to occur in the retina and fluorescein angiography shows choroidal vascular atrophy [19]. Some RP patients even have blood circulation disorders of the body with slower cold-induced maximal flow reduction and longer warm recovery time than non-RP patients [20]. Interestingly, some RP patients have higher levels of plasma ET-1 [7]. Several studies have shown a negative correlation between plasma ET-1 and choroidal thickness or BF in RP patients [20, 21]. Decreased density or BF of the choroidal microvasculature during the physiopathological progression of RP is a distinct symptom in RP patients and in animal models [22]. These studies suggest that ET-1 might exacerbate ischemia of photoreceptors and retinal pigment epithelial (RPE) cells by constricting the choroidal vasculature and thereby decreasing BF.

However, these previous results all lack direct evidence to confirm that ET-1 induces choroidal vasoconstriction, since their means of evaluation were either Doppler flowmetry or spectral-domain optical coherence tomography (OCT) [23, 24], neither of which can detect subtle changes in choroidal microvascular morphology in animal models. Interestingly, using a microvascular corrosion casting technique, we could detect changes in choroidal microvascular densities in small animals, showing them to have lower densities with higher plasma ET-1 or local ET-1 levels in mice, thus implying that ET-1 might lead to a reduction of choroidal BF and therefore to photoreceptor impairment.

Reasons for elevated ET-1 levels in RP patients were not clear yet. However, oxidative stress (OS) and inflammation are important factors also participating in the pathogenesis of RP [25, 26], which may have stimulated vascular endothelial cells and several other kinds of cells to produce and secrete more ET-1 [27]. Inversely, ET-1 was found to be a proinflammatory factor, and ET_A_ or ET_A_/ET_B_ dual receptor blockade attenuated inflammation in mice given a high fat diet [28]. Our data showed upregulated levels of ET-1 in the plasma and ocular tissue of which the direct regulation mechanisms were not clear yet. However, previous studies revealed that inflammation and OS were two pathophysiology features aggravating retinal degeneration in the ocular tissue of rd1 or rd10 mice [29, 30], implying that the expression of ET-1 may be induced by these two factors at least. And, there was a lack of reports about the body pathological changes in rd10 mice. Therefore, exploring these changes of rd10 mice may be an interesting research field which may deepen our understanding of retinal degeneration.

Bosentan, a dual ET_A_ and ET_B_ antagonist, was used to determine the mechanism by which ET-1 acts in the pathological progression of retinal degeneration. In this study, bosentan was found to preserve choroidal microvasculature density and retinal thickness in a RP animal model. Several studies have found that bosentan could prevent the reduction of retinal and choroidal blood flow [12, 31, 32]. ET_A_ is the major type of endothelin receptor on vascular smooth muscle cells. It promotes vasoconstriction via ET-1 binding to ET_A_. Bosentan can weaken this effect and thereby increase vessel diameter [33]. Systematic application of ET_B_ antagonist downregulates ET-1 production by endothelial cells by preventing ET-1 from binding to ET_B_ [34]. Decreased ET-1 levels may amplify the blocking effect. According to our data, bosentan attenuated plasma and RPE/choroid/sclera ET-1 expression in rd10 mice. Bosentan can be administered orally, which means repeated usage and large doses are safe and feasible in animal studies. Finally, inflammation is harmful in the progression of degeneration diseases. ET-1 blockade may inhibit inflammation, which can attenuate the progression, and the reduced inflammation also inhibits ET-1 expression [35, 36].

There is some mystery regarding how bosentan preserves choroidal microvascular densities in rd10 mice. The choroidal capillaries lack vascular smooth muscle which mainly express ETA whereas contain large amounts of endothelial cells expressing high levels of ETB. ET_A_ but have a great many endothelial cells mainly expressed ET_B_. ET_B_ was found to mediate vasodilatation induced by endothelial cells partly by inducing the release of NO. In a rabbit experiment, the choroid vessels constricted after a specific ET_B_ blockade [37] and *in vitro* ocular vessels stimulated by a ET_B_ agonist generated a vasodilation response [38]. So bosentan might weaken the endothelial cells mediated vasodilation by inhibiting ET_B_ which was contrary to our results. However, the dual ET_A_ and ET_B_ antagonist in our research did not show lower choroidal microvascular densities. The persistent increase in choroidal blood flow from medium and large vessel layers might weaken endothelial-mediated microvascular vasocontraction. The choroid consists of many nonvascular smooth-muscle cells presumed to be related to several physiological functions especially for regulating choroid thickness which probably expressed ET_A_ receptors as vascular smooth-muscle cells. Bosentan might dilate capillaries by promoting nonvascular smooth-muscle cells relaxation and thickening the choroid.

Our study has some limitations. We could not fully elucidate the exact mechanism here, which we will further investigate by application of specific ET_A_ and ET_B_ antagonists in RP animal models. Vascular luminal casting cannot represent blood patterns in a live organism. However, ultrahigh-resolution magnetic resonance imaging and advanced laser Doppler can show live dynamic choroidal blood flow in mice. We believe these techniques would advance the research.

In conclusion, we demonstrated herein that plasma ET-1 levels were increased in rd10 mice, implying ET-1 may take part in RP pathogenesis. Furthermore, bosentan, a dual endothelin receptor antagonist, inhibited ET-1-induced choroidal microvascular degeneration and alleviated the progression of RP. These findings provide a new means of investigating the mechanisms of ET-1 on RP in the future.

## Figures and Tables

**Table 1:** Plasma ET-1 levels (pg/ml) in all groups (*n* = 6).

	P14	P21	P28
C57	43.21±24.24	33.52±16.33	42.38±17.53
rd10	58.45±14.32	65.48±24.83	85.89±20.23

## Data Availability

All the data used in this study are available from the corresponding author upon reasonable request.
